# Promise and challenges of dystonia brain banking: establishing a human tissue repository for studies of X-Linked Dystonia-Parkinsonism

**DOI:** 10.1007/s00702-020-02286-9

**Published:** 2021-01-13

**Authors:** Cara Fernandez-Cerado, G. Paul Legarda, M. Salvie Velasco-Andrada, Abegail Aguil, Niecy G. Ganza-Bautista, J. Benedict B. Lagarde, Jasmin Soria, Roland Dominic G. Jamora, Patrick J. Acuña, Charles Vanderburg, Ellen Sapp, Marian DiFiglia, Micaela G. Murcar, Lindsey Campion, Laurie J. Ozelius, Amy K. Alessi, Malvindar K. Singh-Bains, Henry J. Waldvogel, Richard L. M. Faull, Regina Macalintal-Canlas, Edwin L. Muñoz, Ellen B. Penney, Mark A. Ang, Cid Czarina E. Diesta, D. Cristopher Bragg, Geraldine Acuña-Sunshine

**Affiliations:** 1 Sunshine Care Foundation, 5800 Roxas City, Capiz Philippines; 2 Department of Neurosciences, College of Medicine-Philippine General Hospital, University of the Philippines Manila, Manila, Philippines; 3 Department of Neurology, The Collaborative Center for X-linked Dystonia-Parkinsonism, Massachusetts General Hospital, Boston, MA 02129 USA; 4 Stanley Center for Psychiatric Research, Broad Institute, Cambridge, MA 02142 USA; 5 Department of Anatomy with Medical Imaging, Centre for Brain Research, University of Auckland, Auckland, New Zealand; 6 Department of Neurosciences, Makati Medical Center, Makati, Philippines; 7 Department of Pathology, College of Medicine, University of the Philippines, Manila, Philippines

**Keywords:** Neuropathology, Neurodegeneration, Brain, X-Linked Dystonia-Parkinsonism, TAF1, Movement disorders

## Abstract

X-Linked Dystonia-Parkinsonism (XDP) is a neurodegenerative disease affecting individuals with ancestry to the island of Panay in the Philippines. In recent years there has been considerable progress at elucidating the genetic basis of XDP and candidate disease mechanisms in patient-derived cellular models, but the neural substrates that give rise to XDP in vivo are still poorly understood. Previous studies of limited XDP postmortem brain samples have reported a selective dropout of medium spiny neurons within the striatum, although neuroimaging of XDP patients has detected additional abnormalities in multiple brain regions beyond the basal ganglia. Given the need to fully define the CNS structures that are affected in this disease, we created a brain bank in Panay to serve as a tissue resource for detailed studies of XDP-related neuropathology. Here we describe this platform, from donor recruitment and consent to tissue collection, processing, and storage, that was assembled within a predominantly rural region of the Philippines with limited access to medical and laboratory facilities. Thirty-six brains from XDP individuals have been collected over an initial 4 years period. Tissue quality was assessed based on histologic staining of cortex, RNA integrity scores, detection of neuronal transcripts in situ by fluorescent hybridization chain reaction, and western blotting of neuronal and glial proteins. The results indicate that this pipeline preserves tissue integrity to an extent compatible with a range of morphologic, molecular, and biochemical analyses. Thus the algorithms that we developed for working in rural communities may serve as a guide for establishing similar brain banks for other rare diseases in indigenous populations.

**Supplementary Information:**

The online version contains supplementary material available at 10.1007/s00702-020-02286-9.

## Introduction

Brain donation plays a critical role in the study and understanding of human neurodegenerative disease (Kirch et al. 1991; Stopa and Bird 1989; Trujillo Diaz et al. 2018). Creating an effective brain bank that can collect and process research-quality brain tissue requires the coordination of a well-organized, well-funded team that may include clinicians, neuropathologists, scientists, nurses, counselors, logistics managers, outreach coordinators, and other research staff (Cruz-Sanchez and Tolosa 1993; Murphy and Ravina 2003; Trujillo Diaz et al. 2018; Vonsattel et al. 2008a). By categorizing tissue in a way that correlates morphologic findings with clinical diagnoses, research arising from donated brain tissue may lead to the discovery of biomarkers and novel therapeutic approaches to improve overall health outcomes (Trujillo Diaz et al. 2018).

X-Linked Dystonia-Parkinsonism (XDP) is an example of a progressive neurodegenerative disease in need of this approach, as its underlying neuropathology is not well understood and can only be directly assessed in postmortem brain tissue from clinically characterized donors. It is endemic to the island of Panay in the Philippines, with a reported prevalence of 5.74 cases per 100,000 individuals in this region (Pasco et al. 2011), and is thought to have arisen due to a founder mutation in this population (Aneichyk et al. 2018; Wilhelmsen et al. 1998). The clinical phenotype reported most frequently in the literature involves an unusual temporal progression, beginning with hyperkinetic symptoms at early disease stages that over time combine with hypokinetic, parkinsonian features (Evidente et al. 2002; Lee et al. 2002). Recent genetic studies have linked XDP to a hexameric DNA repeat expansion, (CCCTCT)_n_, within a disease-specific insertion of a SINE-VNTR-Alu (SVA)-type retrotransposon in the *TAF1* gene (Bragg et al. 2017; Makino et al. 2007). The length of this repeat tract varies among XDP patients and inversely correlates with the age of disease onset (Bragg et al. 2017; Westenberger et al. 2019). In XDP cell models, the retrotransposon insertion disrupts splicing of *TAF1*, thereby decreasing levels of the full-length transcript (Aneichyk et al. 2018).

Few studies have directly analyzed postmortem XDP brain tissue, reporting an apparently progressive atrophy of the neostriatum in small numbers of cases that came to autopsy at different stages of disease (Goto et al. 2005,2013; Waters et al. 1993). Immunostaining of these specimens suggested that this atrophy represented a selective loss of striosomal medium spiny neurons (MSNs) accompanied by astrogliosis, with apparent sparing of cholinergic interneurons and no other lesions documented in other brain regions (Goto et al. 2005). However, recent neuroimaging of XDP patients and matched control individuals has detected evidence of volume loss not only in the striatum but also the globus pallidus, as well as reduced cortical thickness in frontal and temporal cortices and widespread abnormalities in both grey and white matter (Blood et al. 2018; Bruggemann et al. 2016, 2017; Hanssen et al. 2018). Thus the striatum may not be the only site of dysfunction in XDP, and a complete understanding of the disease pathology will require a broader examination of neuronal subpopulations in human postmortem brain tissue.

To address this need, we developed a brain banking platform in the Philippines to create a repository of XDP brain tissue for characterizing the neuropathology of this disease. The practice of collecting human postmortem brain tissue for studies of the nervous system has been in place for many decades (Cruz-Sanchez and Tolosa 1993; Kretzschmar 2009; Tourtellotte 1970), and multiple reports have documented initiatives to bank CNS tissue for various neurodegenerative disorders, including Parkinson’s disease, Huntington’s disease, Alzheimer’s disease, and amyotrophic lateral sclerosis (Bidaut-Russell et al. 1991; Chan et al. 2020; Cruz-Sanchez et al. 1993; Murphy and Ravina 2003; Ravid and Ferrer 2012; Reynolds and Pearson 1993). In contrast, there is less information about specific strategies for collecting brain tissue from individuals with dystonia. Most descriptions of the neuropathology of dystonia have been based on limited numbers of cases (Paudel et al. 2012; Sharma 2019; Standaert 2011), with very few surveys addressing how individuals with dystonia view brain donation (Kuhta et al. 2011). Furthermore, many of the published consensus standards and operating protocols (Freund et al. 2018; Klioueva et al. 2018; Ramirez et al. 2018; Zielke and Mash 2018) largely reflect the experience of brain banks in metropolitan settings that may not directly translate to non-urban settings. Here we outline the system that we successfully implemented in Panay despite limited availability of medical and laboratory facilities. We summarize the key adaptations needed to meet the logistical challenges of working within this predominantly rural region, as well as the factors influencing brain donation in this population and initial quality control data on postmortem tissue collected with this pipeline. To our knowledge, this initiative represents the first human brain bank established for any disorder in the Philippines and may serve as a roadmap for future initiatives in similar rare disease populations.

## Materials and methods

### Donor recruitment and informed consent

The complete process for identifying and recruiting prospective donors and obtaining informed consent for brain donation is described in detail in the results section. All procedures related to the collection, processing, and use of XDP patient post-mortem brain tissue were approved by institutional review boards at Makati Medical Center (Makati City, Philippines; protocol MMCIRB 2017-134) and Massachusetts General Hospital (Boston, MA, USA; protocol 2016p-000427).

### Tissue processing

After extraction, brains were placed in double Ziploc-type plastic bags and immediately laid in a bucket with wet ice, then transported to the XDP brain bank facilities in Panay and weighed. From each brain, the brainstem and cerebellum were removed, and the corpus callosum was cut through the sagittal plane to divide the hemispheres such that one could be processed as fresh frozen tissue (Vonsattel et al. 2008b) and the other perfused for fixation according to the protocols of the Centre for Brain Research at the University of Auckland (Waldvogel et al. 2006, 2008). The hemisphere processed for fresh frozen tissue was cut in the coronal plane into approximately 16 1-cm thick sections. The cerebellum was divided into four sections along the sagittal plane, and the brainstem was cut in the transverse plane into seven sections corresponding to (1) midbrain; (2–4) upper, middle and lower pons; and (5–7) upper, middle and lower medulla. Sections were photographed and then laid flat on Teflon-coated T316 steel plates (7 × 5.5 × 0.1875 inches; weight = 2 lbs) atop dry ice, with a second plate placed on top of the tissue with additional dry ice so that sections were frozen while pressed gently between the plates. After freezing, sections were individually transferred into labeled zip lock bags, carefully packed into 3-inch high cardboard boxes, and stored at − 80 °C until shipment. Each cardboard box was double-wrapped in sealable biohazard bags and shipped on dry ice to the brain bank facility at the Collaborative Center for X-Linked Dystonia-Parkinsonism (CCXDP) at Massachusetts General Hospital. After arrival at the CCXDP facilities, brain sections were unwrapped, visually inspected, catalogued, and photographed. Tissue was stored at − 80 °C pending further blocking as needed for requested experiments.

### Genotyping

The XDP status of each brain was confirmed by extracting genomic DNA from tissue using the DNeasy™ Blood and Tissue Kit (Qiagen 69504) as recommended. PCR amplification for known haplotype markers was performed as previously described (Bragg et al. 2017; Ito et al. 2016).

### Histology

Small 1.5 cm^2^ blocks of frozen tissue were taken from the BA9 cortical region of each brain. A CM3050S Cryostat (Leica Biosystems) was used to cut 8 μm sections that were mounted on Superfrost Plus™ microscope slides (Fisher Scientific 22-037-246). Slides were allowed to warm to room temperature for 30 s before incubating in 75% ethanol and then distilled water for 30 s each. Mounted sections were covered in 100 μl of HistoGene™ Staining Solution (Thermo Fisher KIT0415) for approximately 30 s and then washed for 30 s each in a series of distilled water, 75% ethanol, 95% ethanol, and 100% ethanol. Slides were incubated in xylene for 5 min and then coverslipped with Permount™ Mounting Medium (Fisher Scientific SP15-100). Tissue was assessed visually on an inverted light microscope with representative images captured at 20 × and 40 × magnification of cortical layer V pyramidal cells.

### RNA extraction

Thirty micrometre cryostat sections were taken from a small block of frozen cortex (BA9 region) from each brain and transferred to microfuge tubes. 500 μl of TRI Reagent (Zymo Research R2050) was added and samples briefly vortexed until the tissue dissolved. RNA was extracted using the Direct-zol™ RNA Miniprep Plus Kit per manufacturer’s instructions (Zymo Research R2072). RNA integrity number (RIN) scores were determined for sample using the Agilent RNA 6000 Pico Kit (5067-1513) on an Agilent 2100 Bioanalyzer as recommended.

### Hybridization chain reaction (HCR)

Eight micrometre cryostat sections were taken from small blocks of frozen BA9 cortex on Superfrost Plus™ microscope slides (Fisher Scientific 22-037-246) and stored at − 80 °C. The slides were warmed to room temperature and post-fixed in 4% paraformaldehyde in PBS for 15 min. Slides were washed 3 × 5 min in 70% ethanol and then soaked in 70% ethanol for 2 hours at room temperature before allowing to dry and demarcating a border around the tissue with a hydrophobic pen. HCR was performed using Molecular Instruments, Inc. (Los Angeles, CA) Multiplex HCR reagents per manufacturer’s instructions (v3.0 protocol). The following HCR probes and matching hairpins were used: Human Reelin (lot PRE441), Human Calbindin-1 (lot PRE481) and Human Aquaporin-3 (lot PRE989). Amplification hairpins used were type B3, B2, and B1 in 546 nm, 488 nm and 647 nm respectively. Following HCR, excess 2 × sodium saline citrate (SSC) was removed with a 200 μl pipette and the slides were cover-slipped using ProLong™ Glass Antifade Mountant (Invitrogen P36981). Images were collected on an Andor CSU-X spinning disk confocal system on a Nikon Eclipse Ti microscope equipped with an Andor iKon-M camera. The images were acquired by an oil immersion objective at 60 ×. Image processing was accomplished using the Nikon NIS elements software package.

### Western blot

Small pieces of frozen BA9 cortical tissue were homogenized on ice for 20 strokes in lysis buffer consisting of 10 mM HEPES (pH 7.2), 250 mM sucrose, 1 mM EDTA, 1 mM NaF, 1 mM Na3VO4, and 1X Complete™ Mini Protease Inhibitor Cocktail (Roche 11836170001). Protein concentrations were determined via Bradford assay (Bio-Rad 5000006). Western blot analysis was performed as previously described (Sapp et al. 2020) to compare protein integrity in XDP tissue lysates relative to control human brain specimens that have been previously characterized (Aronin et al. 1995). Briefly, equal amounts of protein (10 mg) were separated by SDS-PAGE on a 4–12% Bis–Tris NuPage gel (Life Technologies NP0336BOX) and transferred to nitrocellulose. Blots were blocked in 5% milk/Tris-buffered saline (TBS) + 0.1% Tween-20 for 1 hours at room temperature, incubated in primary antibody diluted in blocking buffer overnight at 4 °C, and then incubated in secondary antibody diluted in blocking buffer for 1 hours at room temperature. Bands were visualized using SuperSignal™ West Pico PLUS Chemiluminescent Substrate (Thermo Fisher 34580) and Hyperfilm™ ECL (GE Lifesciences #28906839) using a charge-coupled device (CCD) camera (Alpha Innotech). Antibodies and dilutions used were anti-Neurofilament (1:500, DSHB University of Iowa #2H3), anti-βIII tubulin (1:4000, Sigma T8660), anti-DARPP32 (1:6000, Abcam 40801), anti-GFAP (1:5000, EMD Millipore AB5804), and anti-Iba1 (1:500, Wako 019-19741).

## Results and discussion

### Building an infrastructure to support an XDP brain bank

Our objective was to build a brain banking system that would be centered within a region of the Philippines with a high density of XDP cases to facilitate tissue collection while developing the capacity to distribute specimens to institutions throughout the world for XDP research studies. As with any biobanking initiative, critical starting points were funding for personnel and equipment, as well as institutional support in terms of both laboratory facilities and regulatory governance (Abdaljaleel et al. 2019; Kretzschmar 2009; Rademaker and Huitinga 2018; Ravid 2008; Shepherd et al. 2019). Laying this foundation for an XDP repository presented initial challenges, given that the majority of affected individuals reside within Panay and neighboring areas of the Philippines that together make up the Western Visayas. This greater region, which shares a common dialect with similar socioeconomic and cultural features, comprises approximately 7 million inhabitants, 65% of whom live in rural conditions and 22.4% of whom exist below the annual per capita poverty threshold that is roughly equivalent to USD $460 (Philippine Statistics Authority 2018). Like the rest of the country, residents in this region must pay 53.7% of their health expenses (Chan-On and Sarwal 2017), which can create a particular burden on vulnerable Filipinos, including impoverished XDP patients, that ultimately limits their access to medical care. Thus for donor recruitment and tissue collection we needed to assemble a team in a predominantly rural region without a pre-existing hospital program that centralized care of XDP individuals in a single location.

Towards that objective, we forged critical institutional partnerships at local, national, and international levels. Funding for the XDP brain bank was provided through the affiliation of the Sunshine Care Foundation in Panay with the Collaborative Center for X-Linked Dystonia-Parkinsonism (CCXDP), an international consortium based at Massachusetts General Hospital (Boston, MA, USA) that broadly supports research into the pathophysiology of XDP (Bragg et al. 2019). Within the Philippines, we partnered with Makati Medical Center, a major tertiary care hospital in Manila which provided access to expertise in both pathology and clinical neurosciences. Under their ethics oversight, we also leased private laboratory and office space in Panay from the Health Centrum, a regional hospital in Roxas City, Capiz, which is the province in which XDP cases were first identified (Lee et al. 1976) and which may still contain the highest incidence of disease based on the limited epidemiological data available (Pasco et al. 2011). This facility created an outpost for the brain bank team to process tissue collected in Panay, whereas laboratory space at Makati Medical Center would be used to similarly process specimens from donors in other regions. Because our ultimate goal was to broadly distribute XDP biospecimens for research, we designed a pipeline in which tissue would be collected, processed, and stored short-term in Panay or Manila and then shipped to Massachusetts General Hospital for further blocking, long-term storage, and distribution by CCXDP.

Oversight of this pipeline was set up at multiple levels. Protocols were approved by institutional review boards at both Makati Medical Center and Massachusetts General Hospital; the former directly covered all human subjects interactions, tissue collection, and sharing of biospecimens, whereas the latter covered receipt of de-identified tissue, processing, and secondary distribution for research studies. CCXDP further recruited investigators from well-established brain banks throughout the world who, together with senior members of our investigative team, form an advisory board that reviews requests for tissue from the XDP bank.

### XDP brain bank facilities

The XDP brain bank laboratory suite in Panay was equipped using funds from CCXDP and with materials donated by the Neurological Foundation Human Brain Bank at the University of Auckland (Auckland, NZ). The setup consists of equipment for dissecting, processing, and storing human brain tissue, including: (1) a class II biological safety cabinet and fume hood with perfusion pumps for formalin fixation; (2) a − 80 °C freezer for storing blocked fresh and fixed specimens; (3) a liquid withdrawal CO_2_ gas cylinder with an adapter nozzle for producing dry ice; and (4) bench and storage space for dissection tools and ancillary reagents. The lab is supported by a back-up generator to maintain the uninterrupted flow of electricity to lab equipment at all times.

### Personnel and organizational structure

The XDP brain bank staff consists of a primary coordinator and teams for tissue extraction, bank administration, and outreach. The coordinator serves as the main point of contact and manages all activities. During brain collections, the coordinator is accompanied by the extraction team that includes (1) a pathologist and/or other staff members trained in brain removal; and (2) a genetic counselor and/or other staff members trained to answer questions from next of kin regarding XDP and rationale for brain banking and to obtain informed consent. Although some XDP tissue donors die in regional hospitals, many do so in remote areas serviced only by funeral homes with extremely limited resources. For that reason, the extraction team has been set up as a mobile unit, with a van and driver to transport all equipment required for brain removal in field locations. The administrative team manages consent forms, case reports, death certificates, brain donation documentation, bureau of quarantine requirements, collection of clinical records, and all other business including travel arrangements and purchasing.

To assemble an outreach team, the bank cultivated a network of community stakeholders through multiple educational efforts. Its staff trained lay representatives, some of whom are relatives of XDP patients, to serve as advocates, promoting awareness of the bank’s mission and the importance of XDP research within surrounding communities. The team conducted regular meetings with other community leaders, associations, government officials, funeral home directors, and medical professionals, distributing educational materials about brain banking and giving interviews both in-person and on local radio. The success of these campaigns depended directly on the coordinator’s ability to interface with these different groups, as other banking initiatives have similarly noted (Trujillo Diaz et al. 2018; Waldvogel et al. 2008). The outcome of these efforts was a broad local coalition encompassing the bank’s staff and community members from diverse backgrounds that could work together in identifying prospective tissue donors and educating them and their families about the value of brain donation.

### Donor recruitment and informed consent

The decisions of prospective donors to donate brains to science may be influenced by multiple factors, including (1) knowledge of the process, disease, and/or research; (2) altruistic, religious, and/or spiritual beliefs; (3) opinions of family members; and/or (4) personal experience and time spent with the donation team that engendered trust (Eatough et al. 2012; Lin et al. 2019). The brain bank’s approach to recruitment has focused primarily on the latter, emphasizing time spent with both the prospective donor and family members together, as this practice has been shown to increase the likelihood of donation (Azizi et al. 2006; Schmitt et al. 2001). In these meetings, which often occurred in prospective donors’ homes, the questions most often posed of staff involved (1) the donation process and how it advances XDP research; and (2) concerns over facial disfigurement, given the common local practice of open-casket viewing during wakes. Staff addressed these and all other questions, explaining, in particular, the methods for ensuring that no incision scars would be visible, and provided information on funeral home networks should that assistance be requested.

For donors and families that elected to proceed, the consent was carefully reviewed in English, Tagalog (the national language), or Hiligaynon (the local dialect) as the donor preferred. Donors that provided consent were reminded that it could be withdrawn at any time, and cards with brain bank staff information were given to donors and their families with instructions for contact at the time of death.

### Outline of the brain extraction process

**Fig. 1:**
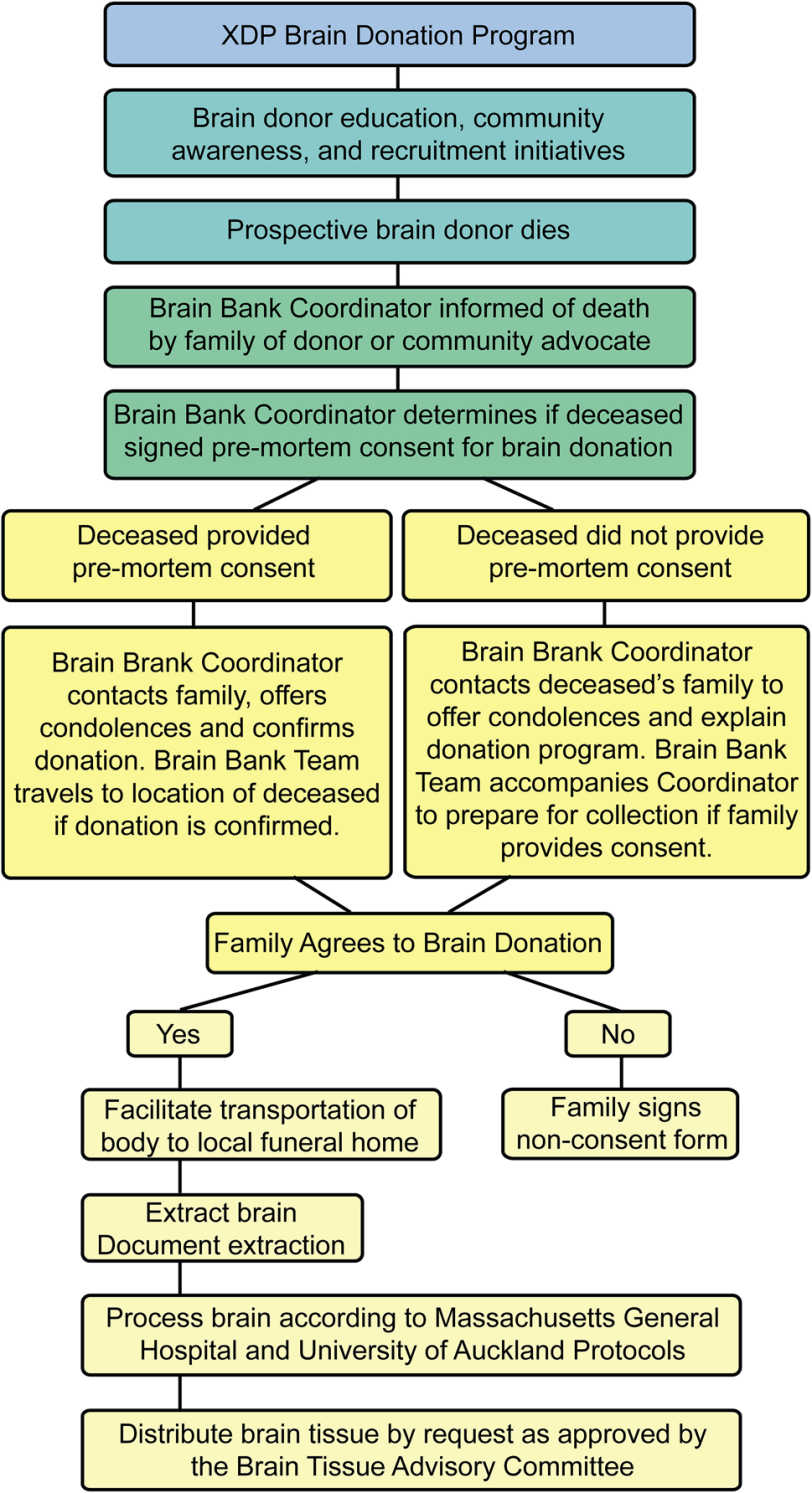
General workflow outlining the process of brain donor recruitment, consent, family engagement, and tissue collection

Figure 1 outlines the general workflow developed by the brain bank team. When a brain donor dies, either the donor’s family or a community advocate immediately contacts the brain bank coordinator. If the deceased signed a consent before death, the coordinator contacts the family to offer condolences and confirm the brain donation. If the deceased did not sign a consent before death, the coordinator contacts the family to offer condolences and assess if the family is open to brain donation. If the family is amenable, the brain bank team proceeds to the deceased’s home to discuss the options. At times, the family’s decision to donate a brain is delayed as other relatives start arriving at the house to join the family in making the decision together. During this time, the brain bank team usually stays with the family, no matter how long, and with its permission, instructs the funeral home to surround the deceased’s head with ice until a decision is made, as other reports have recommended (Trujillo Diaz et al. 2018).

If the family agrees to donate the brain, the team immediately helps transfer the deceased to a funeral home, if not there already, for the brain extraction, which takes 30–60 min. If the family declines brain donation, the team extends its condolences and offers to assist the family with anything else they might need. The team then returns a few weeks later to check on the family and to request a signed acknowledgment indicating that the brain team respected the family’s wishes not to donate the brain. A critical factor to the success of this process is the empathy that the brain bank team is able to show grieving families, which stems in part from the fact that some staff are themselves members of XDP families. To further demonstrate that empathy, the team returns to families that consent to donation on the 1-year anniversary of the passing of the brain donor to honor their contribution to research. These two elements—respecting the wishes of families who choose not to donate and paying tribute to those that do—have been important steps in earning trust for the brain bank team by these communities.

The connection felt among brain bank staff and donor families has instilled an intense sense of mission and dedication among the team. That dedication has been necessary, as the rural nature of the region and geographic distribution of patient families can present extreme challenges to the extraction process. Traveling to where XDP patients die can be grueling. Among the cases collected to date, travel times have ranged from as little as 30 min in each direction to as much as 12 hours each way. This latter example was a 24-hours trip to an adjacent island that involved a 4-hours drive to a ferry dock, a 7-hours ferry ride, and an additional 1-hours drive to the funeral home, arriving at 3 am to perform brain extraction before repeating the entire journey back to the lab suite in Roxas City. In another example, the team flew to Manila to assist with a brain consenting process and extraction there. The physical distance between sites of XDP families can create logistical issues when patients die simultaneously or consecutively. In these cases, the brain team tries to persuade families to use the same funeral home if in sufficient proximity so that brain extractions can be done at the same time. Another recurring challenge is that the region is annually beset by heavy monsoon rains that can wipe away roads, requiring the team to travel at times on dark, unmarked streets. Thus building an XDP brain bank in Panay has not only required outreach strategies tailored to these patient families but also multiple adaptations to the logistical challenges posed by this geographic region.

### Response rate and factors influencing decisions to donate

From June 2016 to June 2020, 80 XDP patients identified by the brain bank staff came to autopsy, of whom 36 (45%) donated brains and 44 (55%) did not (Table 1). Reasons given by the families who agreed to brain donation mirrored data collected by other biobanks, including (1) wanting to advance scientific research to help relatives affected by the disease (Lin et al. 2019); (2) having strong positive relationships with brain bank staff (Eatough et al. 2012); (3) feeling understood during their time of grief and appreciated for the donation (Boyes and Ward 2003; Eatough et al. 2012; Fonseca et al. 2015; Lin et al. 2019); and (4) receiving assistance with funeral arrangements from brain bank staff (Danguilan et al. 2012).

**Table 1:** XDP brain donors vs. non-donors

	Number	Percentage
XDP patient deaths, June 2016–June 2020	80	100
Male donors	35	43.8
Female donors	1	1.2
Total brains collected	36	45
Brains not collected due to		
Logistical constraints despite family consent	7	8.7
Family notified bank too late	18	22.5
Patient chose not to donate pre-mortem	1	1.2
Family chose not to donate post-mortem	15	18.8
Unknown	3	3.8
Total brains not collected	44	55

Some brains were not collected for logistical reasons. In 7 cases (8.7%), families expressed willingness to donate but external factors (monsoons, COVID19 travel restrictions, etc.) prevented brain collection during an appropriate post-mortem interval. In other instances (18 cases, 22.5%), prospective families notified the brain bank staff too late to consider donation as an option. There were also families (20%) that chose not to donate: one in which the prospective donor elected not to participate pre-mortem and 15 in which family members decided against donation (and/or withdrew previously given consent) at the time of death. The importance of family input in decisions surrounding brain donation has been documented by many groups (Eatough et al. 2012; Harris et al. 2013; Lambe et al. 2011; Schmitt et al. 2001; Stevens 1998) and is of particular cultural relevance to Asian populations (Boise et al. 2017a, b; Chan et al. 2020). Reasons cited by XDP families that opted not to donate included (1) lingering concerns over potential facial disfigurement; (2) preference that their relatives be interred “whole;” (3) outstanding questions about the importance of brain banking for research; and (4) grief that precluded making decisions. This feedback aligns with responses others have documented in describing the challenges families may face in making decisions about brain donation at the time of death (Chan et al. 2020; Fonseca et al. 2015). The XDP brain bank staff recognized the importance of achieving family consensus among Filipinos, respecting the decisions of those who chose not to donate, and offering support services in all cases.

### Details of collected cases

Consistent with an X-linked disorder, all but one of the 36 donors were affected males (Table 1). The female brain donor exhibited clinical features of XDP, as may occur due to homozygosity, skewed X-inactivation, or Turner’s Syndrome (Domingo et al. 2014; Evidente et al. 2004; Westenberger et al. 2013). XDP status was confirmed for all cases post-mortem based on PCR amplification of haplotype markers in genomic DNA extracted from brain tissue (Bragg et al. 2017; Ito et al. 2016).

Table 2 lists details of the disease course and post-mortem intervals (PMIs) for the brains collected over the 4-year period. Based on data provided to the brain bank staff, tissue donors had an average age at death of 50.6 ± 8.9 years (range 32–67), an average age of disease onset of 42.6 ± 9.2 years (range 26–59), and an average disease duration of 8.4 ± 4.4 years (range 2–19). This information could not be obtained for some donors, although the calculated means and ranges are consistent with what has been previously reported for XDP (Pasco et al. 2011). For the first 12 brains collected, we reviewed the available clinical data regarding prior history of XDP and symptoms immediately preceding death. Prior to death, most patients had dysphagia, weight loss, and malnourishment, and many patients were bedridden. Cough and difficulty breathing were the symptoms most commonly reported immediately prior to death, suggesting that most deaths were associated with aspiration, pneumonia, and respiratory failure. Further examination of available clinical records revealed no potentially confounding neurologic disease, except one case with evidence of meningitis that was subsequently not banked.

**Table 2:** Brain donor characteristics and post-mortem intervals

	Mean	SDEV	Range
Course of disease (years)			
Age at death	50.6	8.9	32–67
Age at onset	42.6	9.2	26–59
Duration	8.4	4.4	2–19
Brain collection, post-mortem intervals (hours)			
Total time to ice	15.6	8.6	4.8–35.5
Total time to freezing	27.2	15.8	6.8–61.6

We anticipated that one of the most significant challenges in setting up a brain bank within this region would be the PMI, especially in cases where tissue collection had to be performed remotely in field locations under difficult circumstances. Because the collection of the first 12 cases coincided with the setup of the laboratory suite in Panay and the mobile unit, some of these brains had particularly long intervals: the average time from death to extraction (when brains were placed on wet ice) was 23.6 ± 7.56 hours and the average time from death to tissue processing/freezing was 36.8 ± 10.16 hours. As the extraction team’s protocol and laboratory suite were optimized, these intervals were substantially reduced in the subsequent cases with average times to extraction of 10.6 ± 4.40 hours and to freezing of 20.8 ± 15.70 hours. Because the brain bank pipeline included the shipment of frozen tissue from the Philippines to CCXDP facilities at Massachusetts General Hospital, another variable affecting tissue quality was the potential for uncontrolled freeze/thaw events during transport. We, therefore, performed basic quality control measurements to assess general tissue integrity and potential utility in future analyses.

### Assessments of tissue quality

We evaluated measures of basic tissue quality in the first 31 cases collected in Panay and shipped to CCXDP. From these brains, we prepared matched sections of BA9 cortex and performed histologic staining to assess neuronal morphology along with RNA purification for measuring RNA integrity number (RIN) scores. To further probe tissue and RNA quality, we selected a subset of these cases and performed multiplexed hybridization chain reaction (HCR) single-molecule fluorescent in situ hybridization (smFISH) to visualize neuronal subpopulations based on labeling of transcripts. As an index of protein integrity, lysates were prepared from six brains with a range of processing intervals and analyzed via western blot with multiple neuronal and glial markers.

**Fig. 2:**
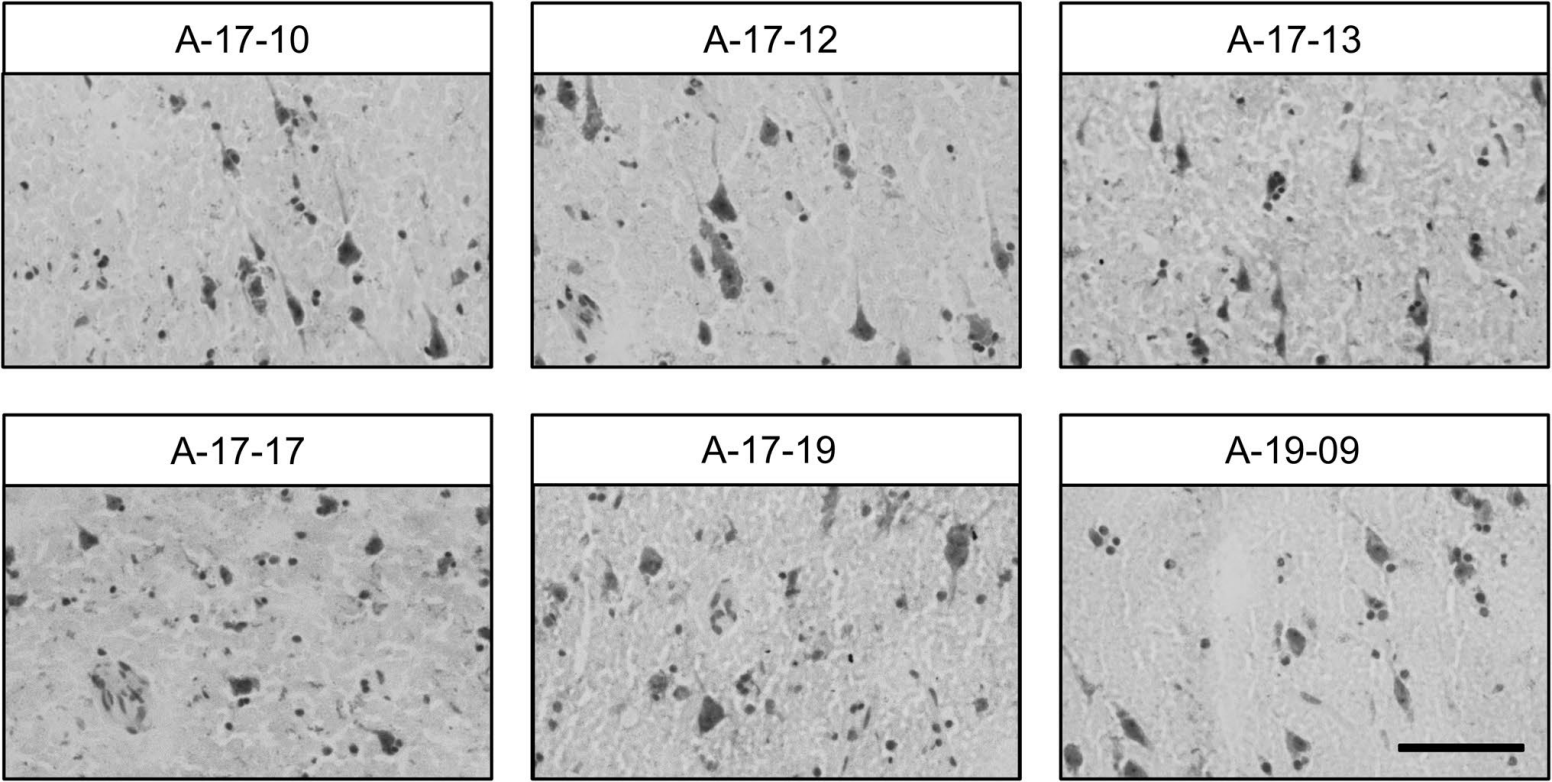
Representative images of BA9 cortex in six XDP brains depicting layer V pyramidal cell neurons visualized via HistoGene™ staining at a final magnification of × 40. Labeled neurons in brains A-17-10, A-17-12, and A-19-09 exhibited typical morphologic features, whereas artifacts of differing severity were present in the other cases, including the porous and/or chicken-wire appearance of tissue that may reflect issues with freezing. Scale bar = 100 μm

Figure 2 depicts sections from six XDP BA9 cortices stained with HistoGene™ (Thermo Fisher) for morphological evaluation, with representative images of layer V pyramidal cells at 40 × magnification demonstrating the range of tissue morphological preservation. Three brains (A-17-10, A-17-12, and A-19-09) exhibited relatively well-preserved morphology in which pyramidal neurons with characteristic large nuclei and prominent nucleoli were easily identified. Two brains, A-17-13 and A-17-19, had freeze artifacts with fractured pyramidal cells and an uneven, porous appearing neuropil. Brain A-17-17 had a more significant freeze artifact that on histology showed holes giving a chicken-wire appearance to the tissue. The remaining 25 brains exhibited variable degrees of tissue preservation that did not correlate with PMI (Supplemental Fig. 1). Brains A-17-10, A-17-12, and A-19-09, which were well-preserved, all had intervals from death to freezing greater than 30 hours, indicating that high-quality tissue can be obtained even after prolonged tissue processing. The freeze artifacts observed may be due to excess water/ice on the tissue surface at the time of freezing; freezing at a suboptimal temperature; or freeze/thaw events during shipping. Additional care is now being taken to remove excess water prior to freezing and to rapidly and uniformly freeze the tissue using pre-cooled Teflon coated aluminum plates pressed between dry ice.

**Fig. 3:**
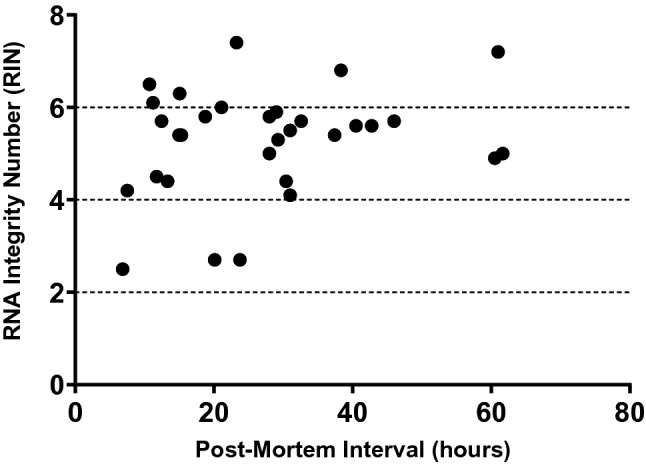
RNA integrity number (RIN) scores vs. post-mortem intervals (PMIs) in 31 XDP brains. Most samples had RIN values ranging from 4.1 to 7.4 suggesting moderate RNA quality with only three samples with a score < 4 indicating significant degradation. Despite the broad range of post-mortem intervals (PMIs) for these brains, there was no correlation between processing times and RNA integrity in these samples (*R*^2^ = 0.1145, *p* = 0.2819)

RNA degradation in post-mortem tissue may be influenced by ante-mortem characteristics, such as metabolic state and agonal period, and post-mortem factors such as PMI, and the extent to which RIN scores directly correlate with any of these factors varies among studies (Birdsill et al. 2011; Harrison et al. 1995; Koppelkamm et al. 2011; Preece and Cairns 2003; Stan et al. 2006; Tomita et al. 2004). The first 31 brains collected by the XDP brain bank had a broad range of processing times, but RIN scores for RNA extracted from BA9 cortex from these cases largely clustered within the same range with no correlation to PMI (Fig. 3). Although three samples had severely degraded RNA based on a score < 4, most had moderately intact RNA with RIN values in the range of 4–7.4, with a mean value of 5.3 ± 1.2, which is generally adequate for most RT-PCR-based experiments (Weis et al. 2007). Furthermore, given that RNA extracted from human post-mortem tissue may often show evidence of some degradation with RIN scores in this range, protocols have recently been optimized for RNA-sequencing of such samples to enable transcriptome profiling under these conditions (Adiconis 2013; Cieslik 2015; Schuierer 2017).

**Fig. 4:**
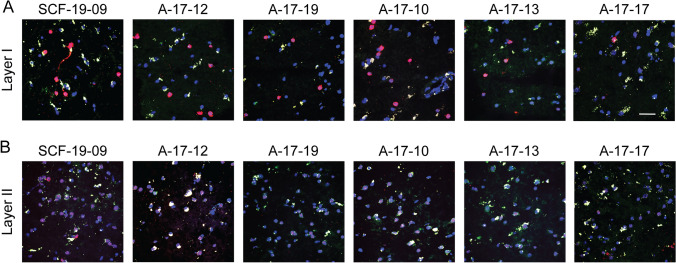
Hybridization chain reaction (HCR) single-molecule fluorescent in situ hybridization (smFISH) analyses of BA9 cortex in six XDP brains. Neurons in cortical layers **a** I and **b** II visualized by multiplexed HCR for Reelin (564 nm Red), Aquaporin-3 (647 nm Magenta), and Calbindin-1 (488 nm Green). Cajal–Retzius neurons in Layer I exhibited robust Reelin expression, while Calbindin-1 and Aquaporin-3 transcripts were less abundant. In layer II, neurons positive for Calbindin-1 and Aquaporin-3 were present with only rare Reelin-positive neurons. In both layers, lipofuscin typical of aged human brain tissue was seen as large yellow/gold puncta in contrast to smaller speckled-appearing Calbindin-1 and Aquaporin-3 signals in green and magenta. Scale bar = 20 μm for all images

A subset of six brains was further analyzed by multiplexed HCR-smFISH which labels target RNAs in situ with fluorescent probes, enabling visualization of defined cell populations within tissue sections (Fig. 4). The assay was designed with probes expected to produce robust labeling (Reelin expression in Cajal–Retzius cells of cerebral cortex layer I); intermediate labeling (Aquaporin-3 expression in neurons and glia of all cortical layers); and weak labeling (Calbindin-1 expression in the neurons of cerebral cortical layer II) (Martinez-Cerdeno and Noctor 2014). All six XDP samples, which had varying degrees of tissue freeze artifact (Fig. 2) and RIN values ranging from 2.7 to 5.7, demonstrated robust Reelin, intermediate Aquaporin-3, and weak Calbindin-1 expression as expected in the appropriate cortical layers. This pattern demonstrates morphological and RNA preservation adequate to enable FISH, suggesting that tissue quality may be compatible with a range of molecular analyses.

**Fig. 5:**
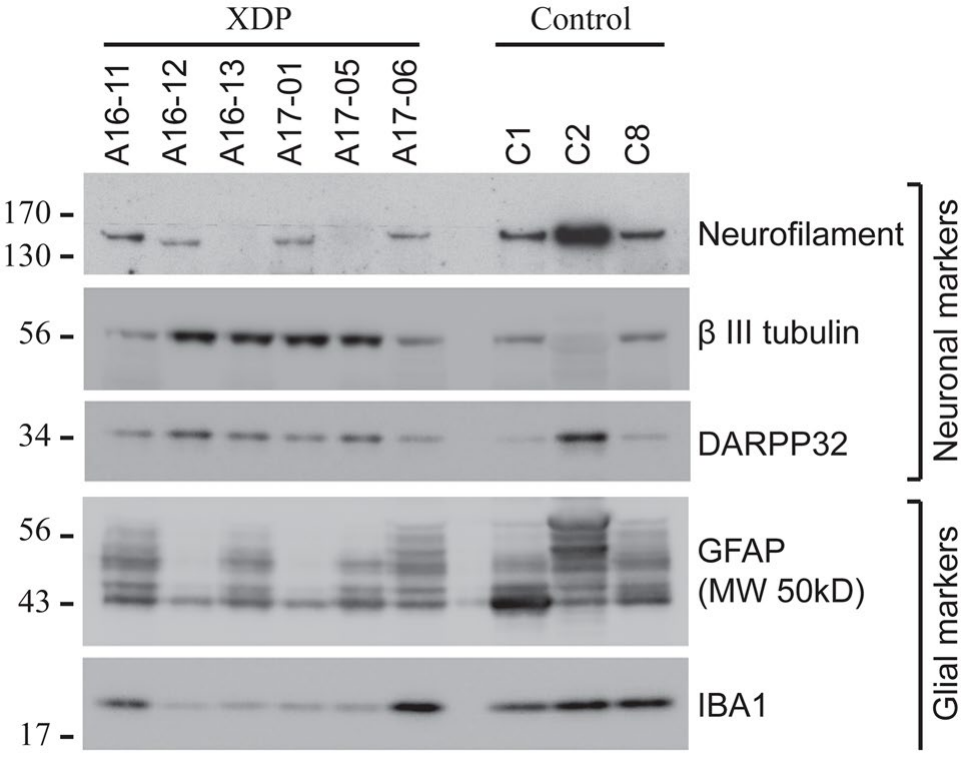
Western blot analysis of lysates from BA9 cortex of six XDP vs. three control brains to assess protein expression of markers of neurons (Neurofilament, βIII-tubulin, and DARPP32), astrocytes (GFAP), and microglia (IBA1). Consistent expression of βIII tubulin, DARPP32, and IBA1 was noted across all samples. Neurofilament could not be detected in XDP samples A-16-13 and A-17-05, and the typical GFAP banding pattern was not observed in XDP samples A-16-12 and A-17-01

Post-mortem protein degradation is sensitive to PMI and the temperature at which the tissue is stored, although the vulnerability of specific proteins to degradation may vary (Ferrer et al. 2007,2008; Siew et al. 2004). Figure 5 depicts a western blot analysis of three neuronal markers and two glial markers in BA9 protein extracts from the same XDP cases analyzed histologically compared to lysates from three human control brains that were collected in other studies (Aronin et al. 1995). All six XDP brains surveyed had detectable levels of neuronal markers, βIII-tubulin and DARPP-32 (Dopamine- and cAMP-Regulated PhosphoProtein, MW 32 kDa), and the microglia marker, Iba1 (Ionized calcium-Binding Adaptor molecule-1). The neuronal marker, neurofilament, however, was not detected in A-16-13 or A-17-05. The astrocyte marker, GFAP (glial fibrillary acidic protein), was detected in most samples as multiple bands between ~ 40 and 50 kDa, but in specimens, A-16-12 and A-17-01, only one or two small, faint bands were observed. GFAP exists in multiple isoforms, and their expression in brain may vary with astrogliosis and/or disease state (Kamphuis et al. 2012; Sereika et al. 2018). We cannot yet determine if the GFAP banding pattern in A-16-12 and A-17-01 reflects differential isoform distribution and/or death-associated proteolysis (Zoltewicz et al. 2012). Nevertheless, the results suggest that protein analysis using this tissue is feasible, as further demonstrated by recent analyses of microglia and astrocytes in these XDP brain samples using immunofluorescence (Petrozziello et al. 2020).

## Conclusions

While brain banking has become widely recognized as a critical tool for studying diseases of the nervous system, many of the reports describing best practices reflect the experience of initiatives in western countries and/or similarly developed metropolitan regions. Implementing these systems in middle/low-income countries and/or rural areas to capture specific populations can present both great opportunities as well as distinct challenges (Akinyemi et al. 2019; Klioueva et al. 2017). XDP is a rare disease that has arisen uniquely within the Philippines, a nation distributed across more than 7000 islands that in some regions include difficult terrains that each year may face powerful tropical storms. The strategies needed to build a bank from tissue donors in these regions, many of whom may be impoverished and live in locales difficult to access, are not necessarily equivalent to those that have worked for banks based in urban universities and hospitals. Our approach has been to systematically educate and build trust within communities of XDP patient families while developing protocols to minimize PMIs as much as possible. The examples discussed here illustrate how lengthy processing times cannot always be avoided, but our initial quality control analyses suggest that tissue collected under such conditions can still be compatible with multiple experimental measures and therefore of great value to future XDP research.

A major consideration for brain banks focused on a specific patient population is the collection in parallel of appropriate control tissue (Harris et al. 2013; Lin et al. 2019), and for neurodegenerative diseases, it has been noted that brain tissue from neurologically healthy individuals can often be difficult to obtain (Schmitt et al. 2001). Our platform collected tissue from XDP brain donors at a rate of 8.75 cases per 6 months over a 4-year period, at a time when the nationwide rate of organ donation in the Philippines has otherwise declined (Chan-On and Sarwal 2017; Danguilan et al. 2012). These observations directly reflect how our outreach campaigns have built widespread community support, and as more XDP patients embrace this mission and express their intent to donate, their unaffected relatives and community members are now beginning to do so as well. We further project that these algorithms for community engagement, donor recruitment, and tissue collection that we developed for XDP may be translatable to other rural populations, thereby serving as guides in future efforts to build similar brain banks for studies of rare disorders.

## Supplementary Information

Below is the link to the electronic supplementary material.

Supplementary file1. Supplemental Figure 1. Representative images of BA9 cortex in 25 XDP brains depicting layer V pyramidal cell neurons visualized via HistoGene™ staining at a final magnification of × 40. Images depict variable degrees of morphologic preservation and artifacts, including prominent chicken-wire appearance in brain A-17-06. Scale bar = 100 μm (TIF 16275 KB)

## Data Availability

Requests for tissue specimens may be directed to xdp@partners.org.
