# Supplementary figures and images for: Promise and challenges of dystonia brain banking: establishing a human tissue repository for studies of X-Linked Dystonia-Parkinsonism
Source: J Neural Transm (Vienna). 2021 Jan 13;128(4):575–87. doi: 10.1007/s00702-020-02286-9 (PMC8099813; doi:10.1007/s00702-020-02286-9)

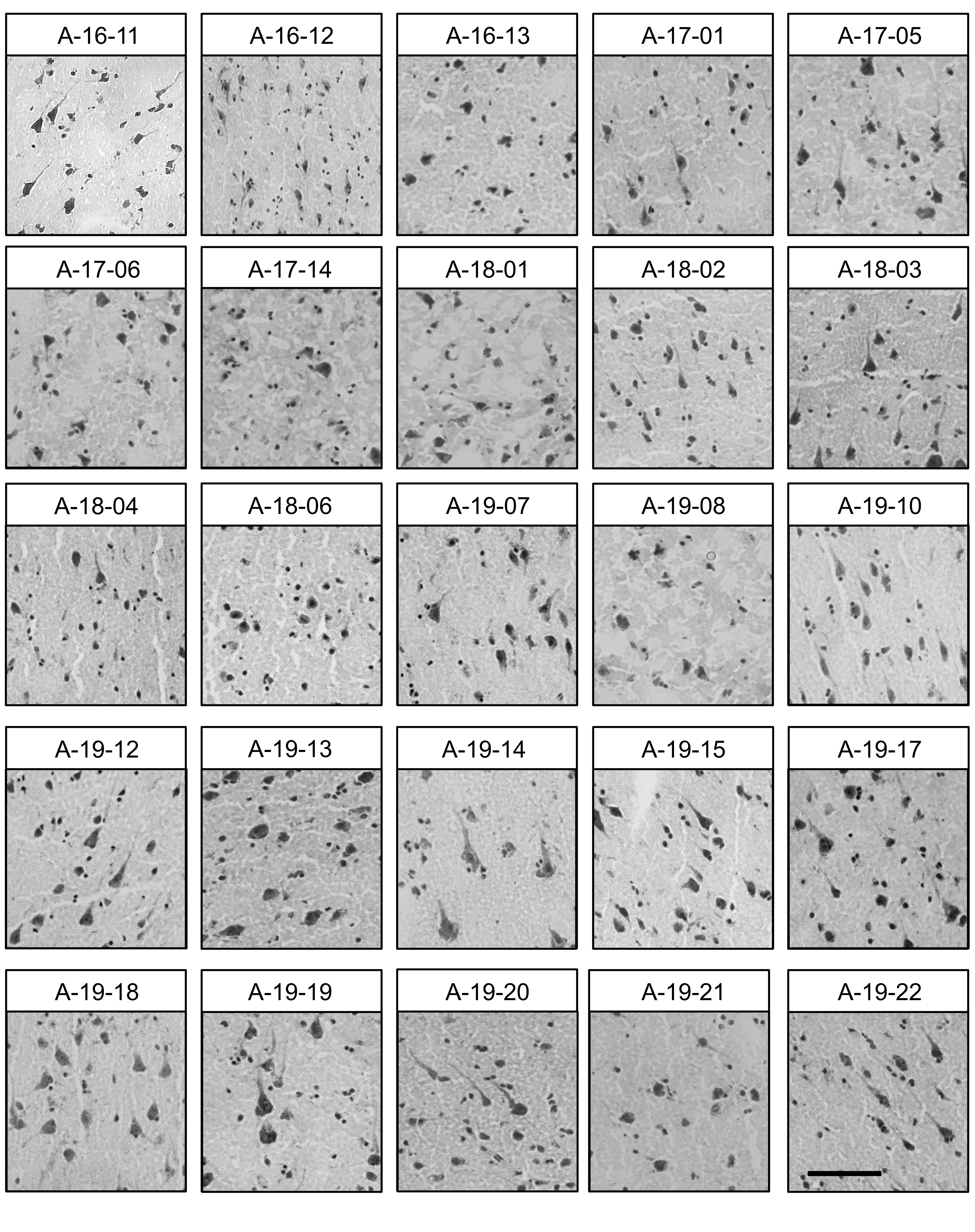

Supplement: Supplementary file 1 — Supplementary file1. Supplemental Figure 1. Representative images of BA9 cortex in 25 XDP brains depicting layer V pyramidal cell neurons visualized via HistoGene™ staining at a final magnification of × 40. Images depict variable degrees of morphologic preservation and artifacts, including prominent chicken-wire appearance in brain A-17-06. Scale bar = 100 μm (TIF 16275 KB) [file 702_2020_2286_MOESM1_ESM.tif]
